# Comparative evaluation of two rapid field tests for malaria diagnosis: Partec Rapid Malaria Test^® ^and Binax Now^® ^Malaria Rapid Diagnostic Test

**DOI:** 10.1186/1471-2334-11-143

**Published:** 2011-05-23

**Authors:** Bernard Nkrumah, Samuel EK Acquah, Lukeman Ibrahim, Juergen May, Norbert Brattig, Egbert Tannich, Samuel Blay Nguah, Yaw Adu-Sarkodie, Frank Huenger

**Affiliations:** 1 Kumasi Centre for Collaborative Research in Tropical Medicine, Kumasi, Ghana; 2 Bernhard Nocht Institute for Tropical Medicine, Hamburg, Germany; 3 Child Health Department, Komfo Anokye Teaching Hospital, Kumasi, Ghana; 4 Department of Clinical Microbiology, School of Medical Sciences, Kwame Nkrumah University of Science and Technology, Kumasi, Ghana; 5 Institute for Transfusion Medicine, Laboratory Medicine and Medical Microbiology, Dortmund, Germany

## Abstract

**Background:**

About 90% of all malaria deaths in sub-Saharan Africa occur in children under five years. Fast and reliable diagnosis of malaria requires confirmation of the presence of malaria parasites in the blood of patients with fever or history suggestive of malaria; hence a prompt and accurate diagnosis of malaria is the key to effective disease management. Confirmation of malaria infection requires the availability of a rapid, sensitive, and specific testing at an affordable cost. We compared two recent methods (the novel Partec Rapid Malaria Test^® ^(PT) and the Binax Now^® ^Malaria Rapid Diagnostic Test (BN RDT) with the conventional Giemsa stain microscopy (GM) for the diagnosis of malaria among children in a clinical laboratory of a hospital in a rural endemic area of Ghana.

**Methods:**

Blood samples were collected from 263 children admitted with fever or a history of fever to the pediatric clinic of the Agogo Presbyterian Hospital. The three different test methods PT, BN RDT and GM were performed independently by well trained and competent laboratory staff to assess the presence of malaria parasites. Results were analyzed and compared using GM as the reference standard.

**Results:**

In 107 (40.7%) of 263 study participants, *Plasmodium sp*. was detected by GM. PT and BN RDT showed positive results in 111 (42.2%) and 114 (43.4%), respectively. Compared to GM reference standard, the sensitivities of the PT and BN RDT were 100% (95% CI: 96.6-100) and 97.2% (95% CI: 92.0-99.4), respectively, specificities were 97.4% (95% CI: 93.6-99.3) and 93.6% (95% CI: 88.5-96.9), respectively. There was a strong agreement (kappa) between the applied test methods (GM vs PT: 0.97; *p *< 0.001 and GM vs BN RDT: 0.90; *p *< 0.001). The average turnaround time per tests was 17 minutes.

**Conclusion:**

In this study two rapid malaria tests, PT and BN RDT, demonstrated a good quality of their performance compared to conventional GM. Both methods require little training, have short turnaround times, are applicable as well as affordable and can therefore be considered as alternative diagnostic tools in malaria endemic areas. The species of *Plasmodium *cannot be identified.

## Background

Half of the world's population is at risk of malaria, with an estimated 243 million cases worldwide out of which 85.6% cases occur in Africa [1]. The World Health Organization (WHO) estimated that 767 000 deaths occurred in Africa in 2008 of which almost 90% were children under five years [1]. Prompt parasitological confirmation by microscopy or alternatively by rapid diagnostic tests (RDTs) is recommended for all patients with suspected malaria before treatment is started [1]. This is to prevent the misuse of antimalarial drugs especially Artemisinin-based combination therapies thereby preventing the possible development of resistance in the parasite to these drugs. Treatment solely on the basis of clinical suspicion should be considered only when parasitological diagnosis is not accessible [1]. Thus, it is of concern that poor diagnostic standards such as the lack of skilled microscopists and inadequate or absence of quality control systems [2] continue to hinder effective malaria control.

Another major contributing factor is that the laboratory diagnosis of malaria has up to now relied nearly exclusively on light microscopy which is a valuable technique when performed correctly but unreliable and wasteful when poorly executed. A better utilization of microscopy and the development of alternative diagnostic techniques could substantially improve malaria control [3]. This study aimed at evaluating and comparing the novel Partec Rapid Malaria Test^® ^(PT) (Partec GmbH, Münster, Germany) and the recently established Binax Now^® ^Malaria Rapid Diagnostic Test (BN RDT) (Binax, Inc., Portland, ME, USA) in malaria diagnosis among children from an endemic area using Giemsa stain microscopy as the reference standard. In a first report we compared PT with GM in a separate collective of patients using a real time PCR assay as reference standard [4]. In this study we focused on the assessment of test result quality and applicability under the field conditions of a rural hospital laboratory.

## Methods

### Study site

The study was conducted at the Agogo Presbyterian Hospital. It is located in the rural Asante Akim North District of the Ashanti Region of Ghana, West Africa and is the principal hospital of the district. The district is located in the eastern part of Ashanti Region and covers a land area of 1,160 km^2 ^with an estimated population of about 142,000 (projection from 2000 Population Census). Over 40% of the population is under 15 years and over 50% are under 20 years of age. The microscopically confirmed incidence of malaria in children under five who reported at the under-five clinic of the Agogo hospital was 265 per thousand in 2008 (unpublished data, Biostatistics Dept, Agogo Presbyterian Hospital).

### Study population

Between June and December 2008 (i.e. from the beginning of the rainy season to the early dry season), 263 cases were enrolled into the study. Samples were obtained from children attending the pediatric clinic of the hospital for the first time with fever or a history suggestive of malaria but without previous antimalarial treatment within two weeks of attending the hospital. Patients diagnosed with malaria were treated with Artesunate - Amodiaquine or Artemether - Lumefantrine as second line treatment representing the Ministry of Health/National Malaria Control Programme approved Artemisinin-based combination therapy for the treatment of uncomplicated malaria in Ghana.

### Ethical Approval

Ethical approval for the study was obtained from the Committee on Human Research, Publication and Ethics (CHRPE) of the School of Medical Sciences, KNUST-Kumasi. After information and appropriate explanations parents or legal guardians of all participants willing to participate in the study had to give their consent by appending their signature or thumbprint to the informed consent form before any study related procedures were done. Internationally accepted Good Clinical and Good Laboratory practices were applied in all procedures.

### Sampling

A maximum of 0.5 ml capillary blood was taken from each child into an EDTA tube labelled with the patient's barcode and pathology number. These samples were examined immediately using the three methods (GM, BN RDT and PT). All three tests were done immediately and examined by well-trained and competent laboratory staff. For GM, the test was examined independently by two expert microscopists in two different rooms with each not knowing the result of the other. In the case where the two microscopic results were discordant, a third expert reader was employed by an independent slide coordinator. The result of the third reader was then considered as the final result. Written results were communicated immediately to the clinicians.

### Giemsa-stained blood film

Thick and thin blood films were prepared using standardized blood volumes of 10 μl and 2 μl, respectively. They were air-dried (thin film was fixed in absolute methanol), heat fixed and both stained with 10% Giemsa working solution for 12 minutes. A malaria blood film was considered negative after 100 high power fields (HPF) has been examined and no parasite observed. If parasites were observed, asexual malaria parasites were counted against 200 white blood cells (WBCs) on the thick film but all parasites in a final HPF were counted even if a count of 200 WBCs had been exceeded. The parasite count per microliter of blood was obtained by using the formula: (Parasite count/200WBC) × Absolute WBC count [5]. If parasite density was more than 100 parasites/field in a thick film, the thin film was used for the count. Upon the observation of asexual malaria parasites, parasitized red blood cells (RBCs) were counted against 1,000 RBCs. The parasite count per microliter of blood was obtained by using the formula: (Parasite count/1000RBC) × Absolute RBC count [6]. The thin film was used for species identification of detected malaria parasites. Absolute WBC and RBC counts were estimated by using the Sysmex KX 21N hematology auto analyzer (Sysmex Corporation, Kobe, Japan). In addition, gametocytes, schizonts and other blood parasites, when present, were reported to the clinicians. Known positive and negative samples were used as controls for freshly prepared Giemsa working solution each day.

### Partec Rapid Malaria Test^®^

The Partec CyScope^® ^(Partec GmbH, Münster, Germany) is a microscope that has two light sources: in addition to the option of normal light microscopy, a fluorescence light detection through incident UV light can be applied. It uses readily-prepared and ready to-use test slides labelled with an unspecific DNA-binding fluorescent dye (4'-6-Diamidino-2- phenylindole (DAPI); emission 443 nm) that detects intraerythrocytic *Plasmodium *DNA [4] resulting in a bright intracellular dot-shaped fluorescence if the RBCs are infected with malaria parasites. The test was performed following manufacturer's instructions [7]. 5 μl of well mixed capillary blood was placed on the dye labeled area of a slide with the patients' pathology number, cover slipped, incubated at room temperature for a minute and observed under the 100× objective (oil immersion) under LED UV light (365 nm). The presence of bright shiny intracellular tiny dots observed under the UV light indicated the presence of malaria parasites in erythrocytes. To prevent the slides from drying out, they were kept in a wet chamber. Parasites counts were done as described in the GM method above. If parasite density was more than 100 parasites/field in a thick film, an approximate count was done by counting a quarter of the field and multiplying by four to get an approximate parasite count per field. This figure was used to calculate the parasite density per microliter. Positive and negative controls were done for each batch of test kits.

### Binax Now^® ^Rapid Diagnostic Test

The BN RDT (Binax, Inc., Portland, ME, USA) is an immunochromatographic membrane assay that uses monoclonal antibodies to detect *P. falciparum *specific histidine rich protein 2 (HRP-2) and a panmalarial aldolase common to all four human pathogenic species in venous and capillary blood specimens [8]. This test was done as described by Wongsrichanalai *et al*. [9]. 15 μl of blood was placed on the purple sample pad; two drops of test reagent were added to the lower absorbent white pad located immediately below the sample pad and four drops to the absorbent white pad up. Lysed blood wicked up to the base of the upper white absorbent pad in a few minutes, when the booklet was closed. The results were read through the viewing window of the device 15 minutes after closing the window. The presence or absences of test lines were used to determine positive and negative results [10] (Table 1). Positive and negative controls were done for each batch of test kits.

**Table 1:** Result interpretation by the Binax Now^® ^RDT

Positive Test		Negative Test		Invalid Test	
**Line**	**Interpretation**	**Line**	**Interpretation**	**Line**	**Interpretation**

C plus T1	Positive for	C only	Negative	No Line	Invalid test.
	*P. falciparum *only				Repeat test with a new kit.

C plus T2	Positive for			T1 or T2 only	Invalid test. Repeat
	*P. vivax *or				test with a new kit.
	*P. malariae *or				
	*P. ovale *or a mix of these. Differentiation of the species is not possible.				

C plus T1	Positive for			T1 and T2 only	Invalid test. Repeat
and T2	*P. falciparum*. It may also represent a mixed infection of				test with a new kit.
	*P. falciparum *with				
	*P. vivax*,				
	*P. malariae*, or				
	*P. ovale*. Differentiation between a *Pf *only infection and a mixed infection containing *Pf *and				
	another malaria species is not possible with this test.				

Source: Binax NOW^® ^reagent package insert, 2007 page 6.

### Statistical analysis

Data were double-entered into a predesigned electronic database using Epi info version 6.04 dfr (Center for Disease Control, Atlanta, GA, USA) and cleaned regularly. Data was exported to *Stata/*SE9.0 statistical software (Stata Corporation, Texas USA) for analysis. Sensitivities, specificities, positive predictive and negative predictive values were determined for the various tests and compared with one another. A p-value less than 0.05 was considered significant in the comparisons. Kappa (k) values expressed the agreement beyond chance [11] and were calculated with a 95% confidence interval. A k-value of 0.21-0.60, 0.61-0.80 and > 0.80 were considered as moderate, good and almost perfect agreement beyond chance respectively. Likelihood ratio tests were included as performance indices in evaluating the test methods.

## Results

### Patient recruitment

From June to December 2008 a total of 263 children (147 males and 116 females) up to 16 years, admitted with fever or a history suggestive of malaria were included in the study with a mean ± SD age of 7.3 years ±3.4 years (range: 3-16 years).

### Giemsa stain microscopy

Overall, GM expert microscopy (Table 2) detected *Plasmodia *in 107 blood films (40.7%) with no discordant readings between the two independent microscopists. Out of these positive films 105 slides (98.1%) were positive with mono-infection of *P. falciparum (Pf)*, of which 2 slides showed *Pf *gametocytes in addition to *Pf *trophozoites, 1 case (0.95%) each was positive with mono-infection of *P. malariae (Pm) *and *P. ovale (Po)*, respectively. 156 films (59.3%) were negative of which 2 films (1.3%) were positive for *Pf *gametocytes only. The geometric mean parasite count was 631.8 parasites/μl (range: 50.0 - 8320.0 parasites/μl) with a turnaround time of 25 minutes per test.

**Table 2:** Results of Giemsa stain, Partec Rapid Test^® ^and Binax Now^® ^RDT

	Positive						Negative		
**Test method**	***Pf *only**	***Pm *only**	***Po *only**	**Mixed Species**	**False Positive**	**Overall**	**Negative**	**False Negative**	**Overall**

GM	105*	1	1	-	-	107	156^§^	-	156
PT^¥^	105	1	1	-	4	111	152	0	152
BN RDT^¥^	52*	-	-	52	10	114	146	3	149

*2 slides with Trophozoites and Gametocytes

^§^2 slides with Gametocytes only

^¥^No Species differenciation

### Partec Rapid Malaria Test^®^

The Partec Rapid Malaria Test^® ^revealed 111 slides to be positive for malaria and 107 slides had concordant results with GM. Thus 4 results were false positive with a mean misdiagnosed parasite count of 32.3 parasites/μl, range: 22.0-43.0 parasites/μl. The geometric mean parasite count was 447.2 parasites/μl (range: 40.0 - 4530.0 parasites/μl) and significantly lower than that obtained from the GM (p < 0.001). The turnaround time per test was 5 minutes.

### Binax Now^® ^Malaria Rapid Diagnostic Test

The BN RDT revealed 114 positive test results: 52 tests showed only two test lines (Table 1: C plus T1) indicative of *P. falciparum *mono-infection whilst 52 tests showed all three test lines (Table 1: C plus T1 and T2) indicative of either *P. falciparum *only or mixed infection of *P. falciparum *and *non-P. falciparum species*. There were 10 false positive and 3 false negative results (Table 2). The BN RDT gave false negative results for one *P. falciparum *infection and in the 2 cases of infection with *P. malariae *and *P. ovale*. Test failures, which were defined as tests that did not show a control band, were not observed with the BN RDT. The turnaround time per test was 20 minutes.

### Sensitivity and Specificity

Compared to the reference standard, the sensitivities of the PT and BN RDT were 100% (95% CI: 96.6-100) and 97.2% (95% CI: 92.0-99.4) respectively, and the specificities were 97.4% (95% CI: 93.6-99.3) and 93.6% (95% CI: 88.5-95.7) respectively (Table 3).

**Table 3:** Performance characteristics of two different methods compared to Giemsa stain microscopy

	Test methods	
	**PT**	**BN RDT**

Sensitivity	100	97.2
(95% CI)	(96.6-100)	(92.0-99.4)
Specificity	97.4	93.6
(95% CI)	(93.6-99.3)	(88.5-96.9)
PPV^1^	96.4	91.2
(95% CI)	(91.0-99.0)	(84.5-95.7)
NPV^2^	100	98
(95% CI)	(97.6-100)	(94.2-99.6)
PLR^3^	39	15.2
(95% CI)	(14.8-103.0)	(8.3-27.6)
NLR^4^	0	0
(95% CI)	(0.0-0.0)	(0.0-0.9)
kappa	0.97	0.9
Std Error	0.06	0.06
p value	< 0.001	< 0.001

^1 ^PPV: Positive predictive value ^2 ^NPV: Negative predictive value

^3 ^PLR: Positive likelihood ratio ^4 ^NLR: Negative likelihood ratio

### Agreement between PT, BN RDT and GM

Table 3 shows the degree of agreement (kappa), with a 95% confidence interval, observed between the different test methods. Overall, there was a high degree of agreement between the test methods and the reference standard.

## Discussion

In this study we assessed the field performance of the PT and BN RDT in a rural Ghanaian hospital laboratory using conventional Giemsa stain thick and thin blood films of pediatric specimens as reference standard. Only few studies in other endemic areas have been conducted specifically in children so far [12-16]. In one study an increased sensitivity of a HRP-2 assay in children compared to adults was demonstrated. This was attributed to lower immunity and possibly less interference by antibodies [17]. Despite this, there is concern that the benefits of parasitological confirmation in children under 5 years may be outweighed by the risks of not treating children with false negative tests [18].

Studies conducted earlier on BN RDT showed a low sensitivity (61.5%) for the detection of pure *P. malariae *and *P. ovale *infections [8]. We found false negative results of BN RDT for only two non-*P. falciparum *infections with low parasite counts (*Pm*: 24 parasites/μl and *Po*: 16 parasites/μl) and thus this might be inadequate to confirm or refute this point. The BN RDT however, has been shown to have a very good sensitivity (100%) for the detection of pure *P. falciparum *infection [9]. *P. falciparum *accounts for almost 99% of malaria infections in the study area (unpublished data, Biostatistics Dept, Agogo Presbyterian Hospital) and, therefore, the low sensitivity of the BN RDT for non-*P. falciparum *malaria may not be a serious cause for concern in this area and most parts of sub-Saharan Africa where malaria infection is predominantly caused by *P. falciparum *[19]. A slight cause for concern might be the number of steps (about 6 steps) involved in performing the test as compared to the four-step test of other RDT formats [20]. However, with adequate training this concern can be addressed. The BN RDT detects the presence of plasmodial antigens HRP-2 and pan-*Plasmodium *aldolase [21]. The detection of 10 false-positive results could be attributed to one main observation made in earlier studies. It is well documented that the HRP-2 antigen can persist up to 28 days after treatment [22,23]. Even though only untreated patients were enrolled in this study, reliability of information obtained from mothers/guardians on this might be questionable. This assumption could have contributed to the increased numbers of false positive tests. In addition this assay appears to be an unsuitable tool for monitoring treatment of malaria as previously discussed by Murray et al [24]. The BN RDT has also been shown to detect parasite levels > 20 parasites/μl for *P. falciparum *and ≤ 100 parasites/μl for *P. vivax *[8]. False negative results could be attributed to the fact that parasite antigen levels were too low to be detected as a result of very low parasitaemia or by semi-immune parasite carriers with low parasitaemia.

Overall the test is simple to perform, rapid (< 15 minutes), easy to interpret, requires less training, needs no laboratory setup and thus it is applicable for field conditions [8,9,21]. The test has also been shown to be heat stable and performs very well at temperatures up to 45°C [25]. On the other hand, it has a poor detection rate for non-*P. falciparum *infections and has a higher false positive rate due to the persistence of the antigen target HRP 2 [8,9,26].

PT uses a fluorescent dye 4'-6-Diamidino-2-phenylindole (DAPI) which detects intracellular double stranded DNA which is present within *Plasmodium*-infected erythrocytes. The bright shiny dots within infected erythrocytes under UV light is extremely characteristic for malaria and has a very high PPV in areas mainly endemic with *P. falciparum *[4]. One major limitation of PT is the disability of specific identification of *Plasmodia *and the differentiation of the species. Compared to GM, PT exhibited four false-positive results which have not been further investigated. PT could also be more sensitive than GM in detecting low number of parasites [4]. The presence of artifacts such as non-specific aggregated DAPI, immature erythrocytes or bacterial cells might have been misinterpreted as plasmodial DNA. The performance characteristics of the tests were very similar as indicated in their sensitivities, specificities, PPV and NPV with very good agreements to the GM reference standard (Table 3). Our study findings correlate well with the results of a study conducted in Sudan for adults using the Partec CyScope^® ^when compared only with conventional Giemsa stained microscopy [27] as well as with our first report comparing this malaria test in another patient's collective [4]. In this first study we could attest to the fact that the PT has a high sensitivity and specificity by referring to the highly sensitive gold standard RT-PCR which, however, cannot be applied in field studies in contrast to the PT assay [4]. Both studies [4,27] confirmed that PT requires very little training and has a short turnaround time of averagely 5 minutes per test. In addition our findings regarding PT performance characteristics and applicability under field conditions were confirmed by a study conducted in Uganda [28]. This study also underlines the disability of specific identification and differentiation of *Plasmodia *species as the disadvantage of PT. However, the characteristic fluorescence of infected erythrocytes has a very high PPV in areas mainly endemic with *P. falciparum*.

Thus we value PT analog to GM thick film as a useful screening method where positive and doubtful results need to be completed by GM thin film examination for confirmation and accurate identification of species later. This strategy takes also into account that Giemsa staining and microscopy is a very elaborate and challenging procedure when performed accurately in a high quality level.

Parasitaemia is used to guide treatment, thus underestimation could have significant ramifications for malaria patients. Parasite counts obtained from PT were significantly lower than those obtained from GM. It is unlikely that parasites are hidden during Giemsa stain microscopy, since adequate preparation of the slide ensures visibility through all planes of focus. The lysis of the red blood cells during the staining process reveals the parasites and ease the detection [29]. The Partec Rapid Malaria Test^® ^employs fluorescence in which red blood cells are not completely lysed, therefore, they may lie on each other or overlap with each other thereby preventing parasites in red blood cells that may be lying beneath other cells from being identified and counted.

The World Health Organization Sexually Transmitted Diseases Diagnostics Initiative (SDI) has developed the ASSURED criteria as a benchmark to decide if tests address disease control needs: Affordable, Sensitive, Specific, User-friendly, Rapid and robust, Equipment-free and Deliverable to end-users [30]. Even though this criteria was developed as a benchmark for the evaluation of RDTs for sexually transmitted infections such as syphilis, it can also be used as a benchmark for the evaluation of RDTs for other diseases such as malaria (Table 4). In rural endemic areas where majority of the people are poor, these tests are affordable enough to be accessed in the health centers and homes.

**Table 4:** Comparison between BN RDT and PT using the ASSURED criteria

	BN RDT	PT^§^
Affordability	$1*	$ 0.5*
Sensitivity (%)	96.5	95.6
Specificity (%)	98	96.7
User-friendly	6 steps	3 steps
Rapid and robust	Yes	Yes
Equipment-free	Yes	No
Deliverable to end-users	Yes	Yes
Minimal Training	Yes	Yes
		

* Data obtained from the manufacturer

^§ ^$ 1300 price for Partec^® ^fluorescence microscope (CyScope^®^)

The accepted level of sensitivity for a rapid diagnostic test in diagnosing malaria is a sensitivity of 95% [31]. Compared to the reference standard, both methods were sensitive and specific enough to be used as diagnostic tools for the diagnosis of malaria in endemic areas.

Both methods require little training and the BN RDT can withstand temperatures up to 45°C [25], making it quite useful for tropical conditions and does not require any equipment. The PT is battery operated, portable and can thus be carried for field work and to places where there may be no regular electricity.

There is the need therefore to expand malaria diagnostic services as part of a greater framework of health system strengthening within resource-limited settings. Increasingly, countries and implementing partners have identified that limited diagnostic capacity represents a major barrier to implementation and sustainability of prevention, treatment and care programs for malaria [32]. The PT and BN RDT, therefore, present as very good tools in the prevention, treatment and care programs for malaria.

## Conclusion

In this study we demonstrated comparable and acceptable performance and operational characteristics of PT, BN RDT and conventional GM with very good agreements with each other. PT and BN RDT require little training only, have very short turnaround times, are applicable under field conditions as well as affordable even in rural areas. Therefore both methods can be considered as alternative diagnostic tools in malaria endemic areas in addition to or instead of GM.

### Recommendations

• This study has found the PT to be a reliable diagnostic tool that is sensitive and specific in diagnosing *falciparum *malaria. Since this is the predominant species causing most mortality and complications in Ghana and sub-Saharan Africa, it is relevant and useful. It is expected that the CyScope^® ^will show similar results for other malaria species especially *P. vivax*, but this could not be ascertained by this study. Further studies are needed to determine its effectiveness in diagnosing other *Plasmodium *species.

• Based on the ASSURED criteria and the need to expand malaria diagnostic services as part of a greater framework of health system strengthening within resource-limited settings [32], the PT and BN RDT can be considered as a point-of-care diagnostic device for resource limited endemic areas.

### Limitations

• In Africa over 70% of malaria cases do not present initially to health facilities but diagnosed and managed at home with traditional remedies or drugs bought from local shops [33]. Patients only attend health centers after self-treatment fails [34]. This might have affected the performance of some of the test methods especially BN RDT.

• The PT is not able to differentiate between *Plasmodium *species.

## Competing interests

The authors declare that they have no competing interests.

## Authors' contributions

BN designed the study protocol, analyzed the data and headed the writing of the protocol. JM, NB, ET, YAS and FH planned and initiated the study and contributed to the writing of the manuscript. SEKA and LI carried out the tests and also contributed to the writing of the manuscript. SBN contributed to the analysis of the results and writing of the manuscript. All authors have read and approved the final manuscript.

## Disclaimer

The findings and conclusions in this paper are those of the authors and do not necessarily represent the views of any of their affiliated Research Institutions.

## Pre-publication history

The pre-publication history for this paper can be accessed here:

http://www.biomedcentral.com/1471-2334/11/143/prepub

## References

[B1] World Malaria Report 2009 http://whqlibdoc.who.int/publications/2009/9789241563901_eng.PDF

[B2] Coleman RE Maneechai N Rachaphaew N Kumpitak C Miller RS Soyseng V Thimasarn K Sattabongkot J Comparison of field and expert laboratory microscopy for active surveillance for asymptomatic Plasmodium falciparum and Plasmodium vivax in western Thailand The American Journal of Tropical Medicine and Hygiene 2002 67 2 141 144 12389937 10.4269/ajtmh.2002.67.141

[B3] Cook GC Malaria: Obstacles and opportunities Transactions of the Royal Society of Tropical Medicine and Hygiene 1992 86 6 699

[B4] Nkrumah B Agyekum A Acquah SEK May J Tannich E Brattig N Nguah SB von Thien H Adu-Sarkodie Y Huenger F Comparison of the Novel Partec Rapid Malaria Test to the Conventional Giemsa Stain and the Gold Standard Real-Time PCR Journal of Clinical Microbiology 2010 48 8 2925 2928 10.1128/JCM.02403-09 20554822 PMC2916594

[B5] Jeremiah ZA Uko EK Comparative analysis of malaria parasite density using actual and assumed white blood cell counts Annals of Tropical Paediatrics 2007 27 1 75 79 10.1179/146532807X170547 17469736

[B6] Frean JA Reliable enumeration of malaria parasites in thick blood films using digital image analysis Malaria Journal 2009 8 1 218 10.1186/1475-2875-8-218 19775454 PMC2761936

[B7] Partec Essential Healthcare 2007 9 10 http://www.partec.com/cms/front_content.php?idcat=39

[B8] Farcas GA Zhong KJY Lovegrove FE Graham CM Kain KC Evaluation of the Binax now (r) ict test versus polymerase chain reaction and microscopy for the detection of malaria in returned travelers The American Journal of Tropical Medicine and Hygiene 2003 69 6 589 592 14740873

[B9] Wongsrichanalai C Arevalo I Laoboonchai A Yingyuen K Miller RS Magill AJ Forney J Gasser RA Jr Rapid diagnostic devices for malaria: field evaluation of a new prototype immunochromatographic assay for the detection of Plasmodium falciparum and non-falciparum Plasmodium The American Journal of Tropical Medicine and Hygiene 2003 69 1 26 30 12932092

[B10] Inverness Medical Binax Now^® ^Malaria Test Kit Product Instructions 2007

[B11] Altman DG Practical statistics for medical research 1991 1 Boca Raton London New York Washington, D.C: Chapman and Hall

[B12] Tarimo DS Minjas JN Bygbjerg IC Malaria diagnosis and treatment under the strategy of the integrated management of childhood illness (IMCI): relevance of laboratory support from the rapid immunochromatographic tests of ICT Malaria Pf/Pv and OptiMal Annals of Tropical Medicine and Parasitology 2001 95 5 437 444 10.1080/13648590120068971 11487366

[B13] Singh N Saxena A Sharma VP Usefulness of an inexpensive, Paracheck^® ^test in detecting asymptomatic infectious reservoir of Plasmodium falciparum during dry season in an inaccessible terrain in central India Journal of Infection 2002 45 3 165 168 10.1016/S0163-4453(02)91055-8 12387772

[B14] Bojang KA The diagnosis of Plasmodium falciparum infection in Gambian children, by field staff using the rapid, manual, ParaSight™-F test Annals of Tropical Medicine and Parasitology 1999 93 7 685 687 10.1080/00034989957925 10715695

[B15] Bechem NN Leke RFG Tietche F Taylor DW Evaluation of a rapid test for histidine rich protein 2 for diagnosis of Plasmodium falciparum infection in Cameroonian children Transactions of the Royal Society of Tropical Medicine and Hygiene 1999 93 1 46 10.1016/S0035-9203(99)90175-X 10492788

[B16] Rimon MM Kheng S Hoyer S Thach V Ly S Permin AE Pieche S Malaria dipsticks beneficial for IMCI in Cambodia Tropical Medicine & International Health 2003 8 6 536 543 10.1046/j.1365-3156.2003.01045.x 12791059

[B17] Fryauff DJ Gomez-Saladin E Purnomo IS Sutamihardja MA Tuti S Subianto B Richie TL Comparative performance of the ParaSight F test for detection of Plasmodium falciparum in malaria-immune and nonimmune populations in Irian Jaya, Indonesia Bulletin of the World Health Organization 1997 75 6 547 552 9509627 PMC2487022

[B18] Interim notes on selection of type of malaria rapid diagnostic test in relation to the occurrence of different parasite species http://www.wpro.who.int/NR/rdonlyres/CF152D7C-25BA- 49E7-86D2-DBEE4E5B5974/0/INTERIMNOTESONMALARIARDTS_ RBM_final2_rev3.pdf

[B19] World Malaria Report 2010 http://www.who.int/malaria/world_malaria_report_2010/malaria2010_summary_keypoints_en.pdf

[B20] Ashley EA Touabi M Ahrer M Hutagalung R Htun K Luchavez J Dureza C Proux S Leimanis M Lwin MM Evaluation of three parasite lactate dehydrogenase-based rapid diagnostic tests for the diagnosis of falciparum and vivax malaria Malaria Journal 2009 8 1 241 10.1186/1475-2875-8-241 19860920 PMC2774865

[B21] van den Broek I Hill O Gordillo F Angarita B Hamade P Counihan H Guthmann JP Evaluation of three rapid tests for diagnosis of P. falciparum and P. vivax malaria in Colombia The American Journal of Tropical Medicine and Hygiene 2006 75 6 1209 1215 17172395

[B22] Humar A Ohrt C Harrington MA Pillai D Kain KC Parasight (R) F test compared with the polymerase chain reaction and microscopy for the diagnosis of Plasmodium falciparum malaria in travelers The American Journal of Tropical Medicine and Hygiene 1997 56 1 44 48 9063360 10.4269/ajtmh.1997.56.44

[B23] Laferi H Kandel K Pichler H False positive dipstick test for malaria New England Journal of Medicine 1997 337 22 1635 1636 9411233 10.1056/NEJM199711273372219

[B24] Murray CK Bennett JW Rapid diagnosis of malaria Interdisciplinary Perspectives on Infectious Diseases 2009 2009 1 7 10.1155/2009/415953 PMC2696022 19547702

[B25] Malaria Rapid Diagnostic Test Performance. Results of WHO product testing of malaria RDTs: Round 1 http://www.finddiagnostics.org/resource-centre/reports_brochures/malaria-diagnostics-report-2009.html

[B26] Bell DR Wilson DW Martin LB False-positive results of a Plasmodium falciparum histidine-rich protein 2-detecting malaria rapid diagnostic test due to high sensitivity in a community with fluctuating low parasite density The American Journal of Tropical Medicine and Hygiene 2005 73 1 199 203 16014858

[B27] Hassan S Okoued SI Mudathir MA Malik EM Testing the sensitivity and specificity of the fluorescence microscope(Cyscope^®^) for malaria diagnosis Malaria Journal 9 1 88 10.1186/1475-2875-9-88 PMC2860357 20356369

[B28] Sousa-Figueiredo JC Oguttu D Adriko M Besigye F Nankasi A Arinaitwe M Namukuta A Betson M Kabatereine NB S JR Investigating portable fluorescent microscopy (CyScope) as an alternative rapid diagnostic test for malaria in children and women of child-bearing age Malaria Journal 2010 9 245 10.1186/1475-2875-9-245 20799940 PMC2940895

[B29] Moody A Rapid diagnostic tests for malaria parasites Clinical Microbiology Reviews 2002 15 1 66 78 10.1128/CMR.15.1.66-78.2002 11781267 PMC118060

[B30] Peeling RW Holmes KK Mabey D Ronald A Rapid tests for sexually transmitted infections (STIs): the way forward Sexually Transmitted Infections 2006 82 suppl 5 v1 17151023 10.1136/sti.2006.024265 PMC2563912

[B31] Towards Quality Testing of Malaria Rapid Diagnostic Tests: Evidence and Methods http://www.wpro.who.int/internet/resources.ashx/RDT/docs/pdf_version/web3_QARDTreport.pdf

[B32] Maputo Declaration 2008 http://www.who.int/diagnostics_laboratory/Maputo-Declaration_2008.pdf

[B33] Amexo M Tolhurst R Barnish G Bates I Malaria misdiagnosis: effects on the poor and vulnerable The Lancet 2004 364 9448 1896 1898 10.1016/S0140-6736(04)17446-1 15555670

[B34] Chandramohan D Jaffar S Greenwood B Use of clinical algorithms for diagnosing malaria1 Tropical Medicine & International Health 2002 7 1 45 52 10.1046/j.1365-3156.2002.00827.x 11851954

